# Clinical pitfalls and serological diagnostics of MuSK myasthenia gravis

**DOI:** 10.1007/s00415-022-11458-4

**Published:** 2022-11-17

**Authors:** Young Nam Kwon, Mark Woodhall, Jung-Joon Sung, Kwang-Kuk Kim, Young-Min Lim, Hyunjin Kim, Jee-Eun Kim, Seol-Hee Baek, Byung-Jo Kim, Jin-Sung Park, Hung Youl Seok, Dae-Seong Kim, Ohyun Kwon, Kee Hong Park, Eunhee Sohn, Jong Seok Bae, Byung-Nam Yoon, Nam-Hee Kim, Suk-Won Ahn, Kyomin Choi, Jeeyoung Oh, Hyung Jun Park, Kyong Jin Shin, Sanggon Lee, Jinseok Park, Seung Hyun Kim, Jung Im Seok, Dae Woong Bae, Jae Young An, In Soo Joo, Seok-Jin Choi, Tai-Seung Nam, Sunyoung Kim, Ki-Jong Park, Ki-Han Kwon, Patrick Waters, Yoon-Ho Hong

**Affiliations:** 1 Department of Neurology, Seoul National University Seoul Metropolitan Government Boramae Medical Center, Seoul, Republic of Korea; 2 Department of Neurology, Biomedical Research Institute, Seoul National University Hospital, Seoul, Republic of Korea; 3 Department of Medicine, Seoul National University College of Medicine, Seoul, Republic of Korea; 4 Oxford Autoimmune Neurology Group, Nuffield Department of Clinical Neurosciences, Neuroimmunology Group, University of Oxford, John Radcliffe Hospital, Level 5, West Wing, Headley Way, Oxford, OX3 9DU UK; 5 Department of Neurology, Seoul National University College of Medicine, Neuroscience Research Institute, Seoul National University Medical Research Council, 20 Boramae-Ro 5-Gil, Dongjak-Gu, Seoul, 07061 Republic of Korea; 6 Department of Neurology, Asan Medical Center, University of Ulsan College of Medicine, Seoul, Republic of Korea; 7 Department of Neurology, College of Medicine, Ewha Womans University, Seoul, Republic of Korea; 8 Department of Neurology, Korea University College of Medicine, Korea University Anam Hospital, Seoul, Republic of Korea; 9 Department of Neurology, School of Medicine, Kyungpook National University, Kyungpook National University Chilgok Hospital, Daegu, Republic of Korea; 10 Department of Neurology, Dongsan Hospital, Keimyung University School of Medicine, Daegu, Republic of Korea; 11 Department of Neurology, Pusan National University Yangsan Hospital, Yangsan, Republic of Korea; 12 Department of Neurology, Uijeongbu Eulji Medical Center, Eulji University School of Medicine, Uijeongbu, Republic of Korea; 13 Department of Neurology, Seoul Medical Center, Seoul, Republic of Korea; 14 Department of Neurology, Chungnam National University College of Medicine, Chungnam National University Hospital, Daejeon, Republic of Korea; 15 Department of Neurology, Kangdong Sacred Heart Hospital, Hallym University College of Medicine, Seoul, Republic of Korea; 16 Department of Neurology, Seoul Paik Hospital, Inje University College of Medicine, Seoul, Republic of Korea; 17 Department of Neurology, Dongguk University Ilsan Hospital, Goyang, Republic of Korea; 18 Department of Neurology, Chung-Ang University Hospital, Chung-Ang University College of Medicine, Seoul, Republic of Korea; 19 Department of Neurology, Konkuk University Medical Center, Konkuk University School of Medicine, Seoul, Republic of Korea; 20 Department of Neurology, Gangnam Severance Hospital, Yonsei University College of Medicine, Seoul, Republic of Korea; 21 Department of Neurology, Haeundae Paik Hospital, Inje University College of Medicine, Busan, Republic of Korea; 22 Department of Neurology, Hanyang University College of Medicine, Seoul, Republic of Korea; 23 Department of Neurology, School of Medicine, Catholic University of Daegu, Daegu, Republic of Korea; 24 Department of Neurology, College of Medicine, St. Vincent Hospital, The Catholic University of Korea, Suwon, Republic of Korea; 25 Department of Neurology, Ajou University School of Medicine, Suwon, Republic of Korea; 26 Department of Neurology, Chonnam National University Medical School, Gwangju, Republic of Korea; 27 Department of Neurology, University of Ulsan College of Medicine, Ulsan, Republic of Korea; 28 Department of Neurology, College of Medicine, Gyeongsang National University, Gyeonsang Institute of Health Science, Jinju, Republic of Korea; 29 Department of Neurology, Hallym University Dongtan Sacred Heart Hospital, Hallym University College of Medicine, Hwaseong, Republic of Korea

**Keywords:** Seronegative myasthenia gravis, Anti-MuSK antibody, ELISA, Cell-based assay, Radioimmunoprecipitation assay

## Abstract

**Background:**

We aimed to evaluate the diagnostic accuracy of enzyme-linked immunosorbent assay (ELISA) for anti-muscle specific tyrosine kinase (MuSK) antibody (Ab) in a large cohort of anti-acetylcholine receptor (AChR) Ab-negative generalized myasthenia gravis (MG), and also to investigate clinical contexts for the diagnosis of MuSK MG.

**Methods:**

A retrospective study of 160 patients with a clinical suspicion of AChR Ab-negative generalized MG was performed. The serum samples were tested for anti-clustered AChR Ab by cell-based assay (CBA), anti-MuSK Ab by ELISA, CBA and/or radioimmunoprecipitation assay (RIPA). Clinical data were compared between anti-MuSK Ab-positive MG and double seronegative (AChR and MuSK) MG groups.

**Results:**

After excluding non-MG and clustered AChR Ab-positive patients, we identified 89 patients as a cohort of AChR Ab-negative generalized MG. Anti-MuSK Ab was positive by ELISA in 22 (24.7%) patients. While CBA identified five additional anti-MuSK Ab-positive patients, the results of ELISA were mostly consistent with CBA and RIPA with Cohen’s kappa of 0.80 and 0.90, respectively (*p* < 0.001). The most frequent differential diagnosis was motor neuron disease particularly of bulbar onset which showed remarkably overlapping clinical and electrophysiological features with MuSK MG at presentation.

**Conclusion:**

While confirming the highest sensitivity of CBA for detecting anti-MuSK Ab, our results highlight the clinical pitfalls in making a diagnosis of MuSK MG and may support a diagnostic utility of MuSK-ELISA in clinical practice.

## Introduction

Acquired myasthenia gravis (MG) is an autoimmune disorder of the neuromuscular junction, caused in most patients by antibodies (Ab) to the muscle nicotinic acetylcholine receptor (AChR). Anti-muscle-specific tyrosine kinase (MuSK) Ab are detected in about 1–10% of all MG patients, with varying regional prevalence [1–3]. Anti-MuSK IgG4 Ab block interaction with low-density lipoprotein receptor-related protein 4 (LRP4), which interferes with MuSK activation and AChR clustering [4]. Although there have been significant advancements in understanding clinical and pathophysiological features of MuSK MG, a diagnosis of this MG subtype may be challenging due to its often atypical clinical manifestations [2]. The unusual clinical features include predominantly regional involvement of bulbar and respiratory muscles, facial and tongue muscle atrophy, and poor response to acetylcholinesterase inhibitors or even cholinergic hypersensitivity, and lower diagnostic yield of electrophysiological tests such as repetitive nerve stimulation (RNS). Accumulating knowledge on the clinical characteristics has contributed significantly to the improvement of diagnosis of MuSK MG. However, a clinical diagnosis of this MG subtype is still often challenging particularly without serological confirmation of anti-MuSK Ab, which may lead to delayed diagnosis and poor treatment outcome [2, 5, 6].

For detection of anti-MuSK Ab, there are three different laboratory techniques currently available which include the live cell-based assay (CBA), radioimmunoprecipitation assay (RIPA), and enzyme-linked immunosorbent assay (ELISA). RIPA is the most commonly used test with almost 100% specificity [7], and CBA was demonstrated to provide a higher sensitivity [8, 9]. Although CBA and RIPA are considered as the gold standard for anti-MuSK Ab detection, they involve either radioactivity or genetically engineered cells which may not be easily available for clinical practice in many regions of the world. As an alternative, there are commercially available ELISA kits for detecting anti-MuSK Ab. However, the diagnostic accuracy of MUSK-ELISA has not been formally tested in a large cohort, and its current use is still limited for the research purpose.

We have reported on the comprehensive autoantibody profiles (MuSK, LRP4, clustered AChR) in patients with anti-AChR Ab-negative generalized MG in South Korea [10]. Following the study, we have continued to collect clinical data and serum samples of the patients with anti-AChR Ab-negative generalized MG. This provided us an opportunity to evaluate the diagnostic accuracy of ELISA for anti-MuSK Ab compared to CBA and RIPA. We also aimed to investigate the clinical contexts (clinical features and differential diagnoses) in which the test for anti-MuSK Ab is requested in a real-world setting.

## Methods

### Patients

This is a retrospective multi-center cohort study. Clinical data and serum samples of adult patients with a high index of suspicion for anti-AChR Ab-negative generalized MG were collected from 26 general hospitals in South Korea between January 2014 and January 2019. Data were entered into a standard case report form designed to record the clinical and laboratory features of the patients. Seronegative generalized MG was diagnosed based on 1) the clinical diagnosis of generalized MG, i.e., the presence of exertional weakness which may affect ocular, limb, axial, bulbar or respiratory muscles but not confined to ocular muscles, and 2) negative result for anti-AChR Ab by RIPA. The results of ancillary diagnostic tests such as low-frequency repetitive nerve stimulation (RNS) and pharmacological test (e.g., neostigmine) may support the diagnosis of MG, but the absence of abnormalities does not rule out the diagnosis considering their low sensitivity in MuSK MG [11].

Collected data were reviewed and assessed for inclusion by two authors (YN Kwon, YH Hong). If there is diagnostic uncertainty, additional data on the disease course and treatment response during clinical follow-up of at least 6 months were requested, and the final diagnosis was reassessed. Disease severity was evaluated by the Myasthenia Gravis Foundation of America (MGFA) clinical classification [12] and the Myasthenia Gravis composite scale (MGCS) [13]. The ocular form at onset was defined as purely ocular manifestations within one month after the symptom onset. Patients with a final diagnosis other than MG in the initial cohort and those with AChR Ab-positive MG were used as disease control to evaluate the specificity of MuSK-ELISA test.

Serum samples were stored at − 80 °C at the central laboratory of the Seoul Metropolitan Government Boramae Medical Center. This study was approved by the local ethics committee of Seoul National University, Seoul Metropolitan Government Boramae Medical Center (IRB 16-2014-29). All patients provided written informed consents.

### Antibody testing

All serum samples were tested for anti-MuSK Ab using a commercial ELISA kit (IBL International GmbH, Hamburg, Germany). Quantitative and qualitative results were determined by use of a standard curve and the cut-off control, respectively. The standard curve was fit by four-parameter logistic regression algorithm, and the cut-off index (COI) was calculated from the mean optical density (OD) of the sample divided by the mean OD of the cut-off standard. Samples with COI over 1 were considered to be positive. Each sample and standard were tested in duplicates.

A subset of serum samples were tested for the antibodies to MuSK by CBA and RIPA, and for the antibodies to clustered AChR by CBA at the Autoimmune Neurology Diagnostic Laboratory, Nuffield Department of Clinical Neurosciences, John Radcliffe Hospital, Oxford, UK [10]. All Ab testing was performed blinded to the clinical information and the results of ELISA. Measurement of Ab binding in CBA was performed by indirect immunofluorescence, as previously described [14, 15]. Results were measured by two observers on a nonlinear visual scale from 0 to 4 with the mean result given. A score of less than 1 was considered to be negative, and scores from 1 to 4 were considered to be positive with 1 weak positive and 4 strong positive.

### Statistical analysis

We used Student’s *t* test and Chi-squire test (or Fisher’s exact test as appropriate) to analyze differences between groups. The one-way analysis of variance followed by Tukey test, and the Chi-square followed by Fisher’s exact test were used for multiple group comparisons. A binary logistic regression analysis was performed to identify clinical features associated with MuSK MG. The variables with a *p* value < 0.2 in the univariable analyses were included in the multivariable model. The variance inflation factor (VIF) was calculated for each independent variable, and the variables with VIF of over 10.0 were excluded from the multivariable analysis. The final model was developed using a backward selection method. Cohen’s kappa was calculated to evaluate the agreements of the results between ELISA, CBA and RIPA. The Spearman rank-correlation test was used to assess the correlation between the results of Ab assays. For all tests, *p* values were two-sided, and the significance level was set at 0.05. Statistical analyses were performed using SPSS software (version 23 for Windows; SPSS, Chicago, IL, USA) or the GraphPad Prism software (version 5.0; GraphPad Software Inc., La Jolla, CA, USA).

### Data availability

The data that support the findings of this study are available on request from the corresponding author. The data are not publicly available due to privacy or ethical restrictions.

## Result

### Clinical diagnosis

**Fig. 1:**
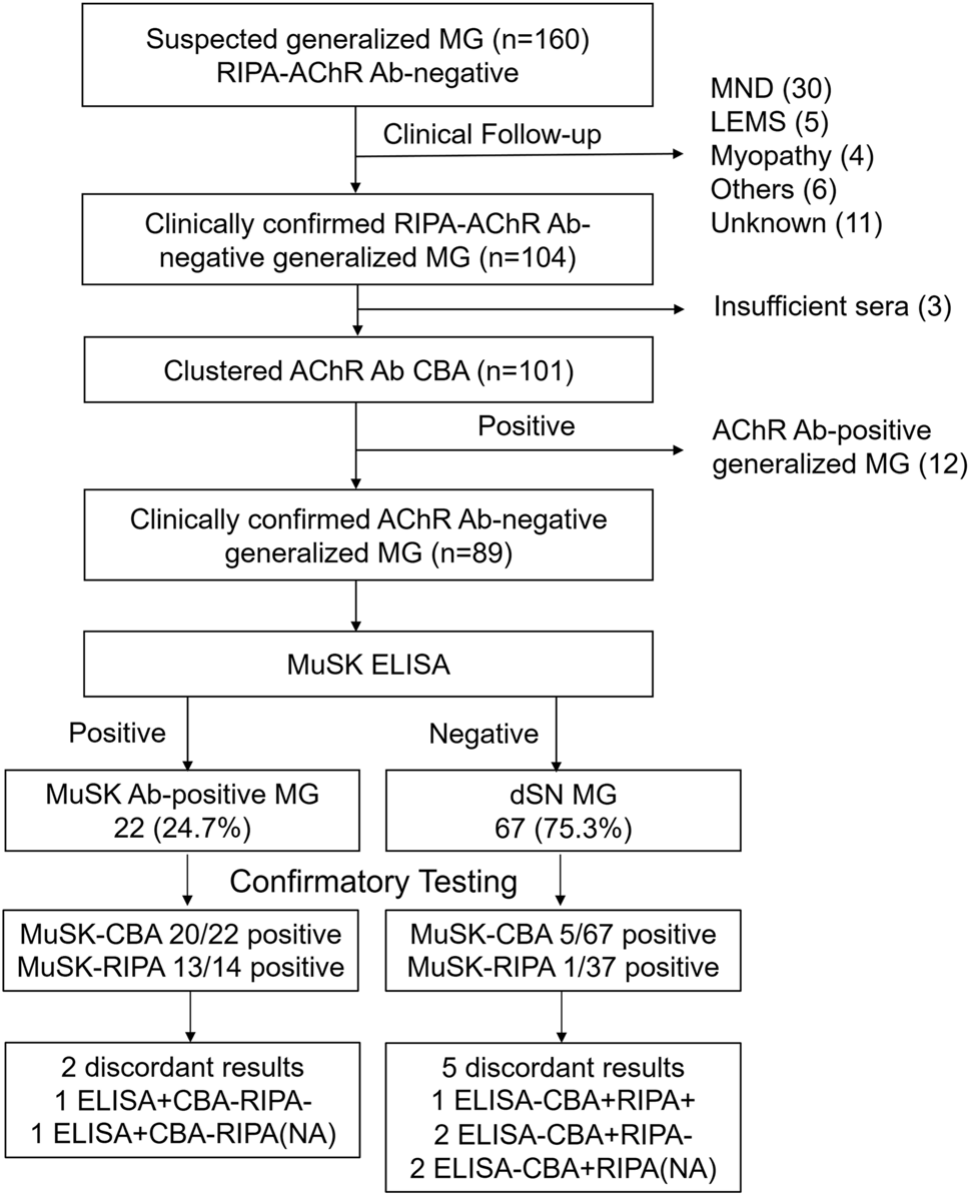
Study flow diagram. The initial cohort consisted of 160 patients with clinically suspected AChR Ab-negative (RIPA) generalized MG. Of these, 104 patients were confirmed clinically, and 89 were finally identified as AChR Ab-negative generalized MG, excluding those seropositive for clustered AChR Ab in CBA. Following MuSK-ELISA, confirmatory antibody testing was performed using CBA and RIPA. *Ab* antibody, *AChR* acetylcholine receptor, *CBA* cell-based assay, *dSN-MG* double seronegative generalized myasthenia gravis, *ELISA* enzyme-linked immunosorbent assay, *MuSK* muscle-specific tyrosine kinase, *NA* not available, *RIPA* radioimmunoprecipitation assay

Clinical data and serum samples were collected from 160 patients with suspected RIPA-AChR Ab-negative generalized MG (Fig. 1). After a review of the clinical data, we excluded patients with non-MG diagnosis and identified 104 patients (65.0%) as RIPA-AChR Ab-negative generalized MG. Diagnoses of the other 56 patients were as follows: 30 motor neuron disease (MND), 5 Lambert-Eaton myasthenic syndrome (LEMS), 3 myopathy, 1 muscular dystrophy, 1 Guillain–Barré syndrome, 1 post-polio syndrome, 1 thyroid-associated ophthalmopathy, 1 atypical parkinsonism, 1 frontotemporal dementia, 1 conversion disorder and 11 unknown.

We tested clustered AChR Ab by CBA in all but 3 patients of the initial cohort, and excluded 12 seropositive patients for the final cohort of AChR Ab-negative generalized MG.

### Serological tests for anti-MuSK antibody

All AChR Ab-negative generalized MG patients (*n* = 89) were tested for the antibodies to MuSK by ELISA and CBA. Samples with sufficient volume were also tested by RIPA (*n* = 51).

**Fig. 2:**
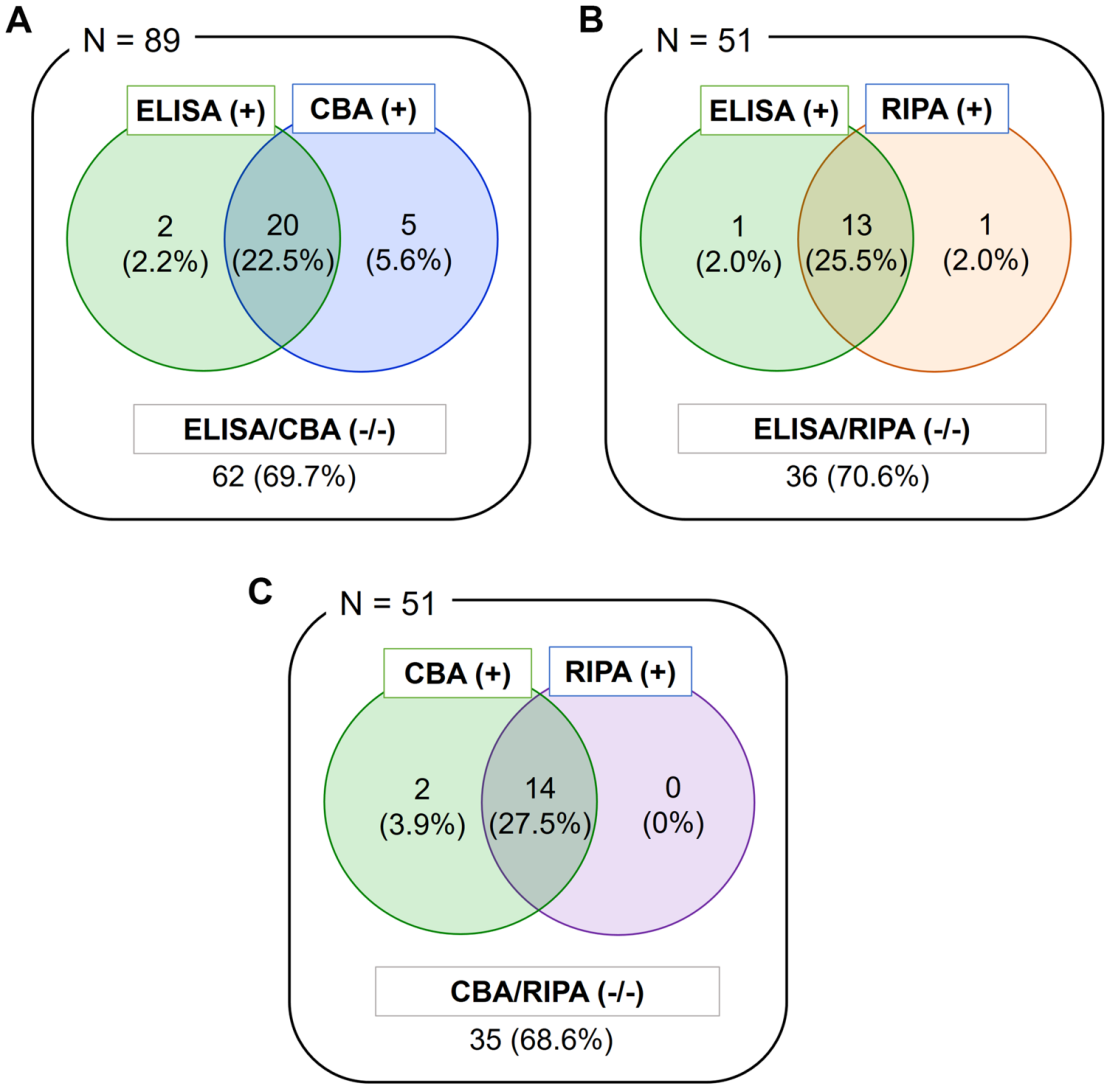
Venn diagram of Serological test results for anti-MuSK antibody. *AChR* acetylcholine receptor, *CBA* cell-based assay, *ELISA* enzyme-linked immunosorbent assay, *MG* myasthenia gravis, *MuSK* muscle-specific tyrosine kinase, *RIPA* radioimmunoprecipitation assay

**Fig. 3:**
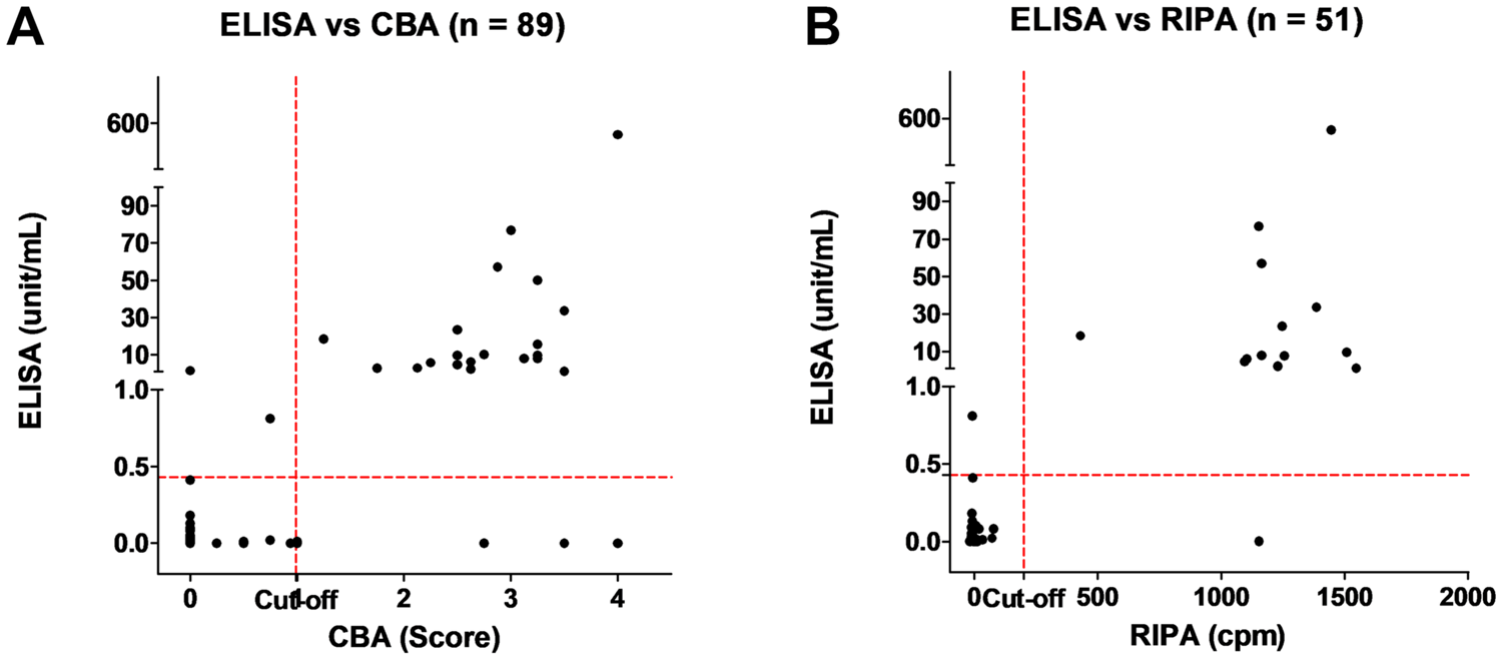
Correlations of anti-MuSK antibody concentration in ELISA with CBA score (**A**) and RIPA value (**B**). *CBA* cell-based assay, *cpm* counts per minute [16], *ELISA* enzyme-linked immunosorbent assay, *RIPA* radioimmunoprecipitation assay

MuSK Ab was positive in 22 patients (24.7%) by ELISA, 25 patients (28.1%) by CBA, and 14 of 51 patients (27.5%) by RIPA (Fig. 2). The results of ELISA were in good agreement with those of CBA and RIPA, with Cohen's kappa of 0.80 (0.66–0.94) and 0.90 (0.77–1.0), respectively (95% CI, *p* < 0.001 for both). There were significant correlations of MuSK Ab concentrations in ELISA with CBA scores and RIPA values [16] (*r* = 0.51, 0.44, *p* < 0.001 for both; Fig. 3).

The results of ELISA and CBA/RIPA were discordant in seven patients. Two patients were positive in ELISA but negative in CBA (RIPA negative in one and not tested in the other one), while five patients were negative in ELISA but positive in CBA (RIPA positive in one, negative in two, not tested in two).

The specificity of MuSK ELISA was 0.95 (95% CI 0.87–0.99) based on the results from 63 serum samples of disease control which consisted of 45 patients diagnosed with a disease other than MG in the initial cohort (MND 30, LEMS 5, Myopathy 4, Others 6) and 18 patients with RIPA-AChR Ab-positive MG. Three of these (3/63) were marginally positive in MuSK ELISA: two with MND and one with LEMS. Confirmatory testing (RIPA and CBA) was performed in 12/63 disease controls and they were all negative (including the MuSK ELISA-positive LEMS patient).

### Clinical characteristics

To investigate the clinical characteristics of MuSK MG in a context of AChR Ab-negative generalized MG, we classified our cohort of 89 patients into 2 groups according to the presence of anti-MuSK Ab. If anti-MuSK Ab was positive in any of the three assays, it was defined as MuSK MG, and if not, it was defined as double seronegative MG. Comparisons between the MuSK MG vs. double seronegative MG groups revealed significant differences regarding the sex ratio (85.2% vs. 43.5%, *p* < 0.001), MGFA bulbar classification at presentation (70.4% vs. 40.3%, *p* = 0.012), myasthenic crisis (40.7% vs. 11.3%, *p* = 0.003), and MGCS score (mean 9.8 vs. 6.1, *p* = 0.046) (Table 1). In comparison to MND patients, those with MuSK MG were younger at symptom onset (mean 48.2 years vs. 62.4 years, *p* < 0.001), predominantly female (85.2% vs. 46.7%, *p* = 0.005), and had more frequently ocular manifestations (25.9% vs. 0.0%, *p* = 0.004) and myasthenic crisis or acute worsening events (40.7% vs. 3.8%, *p* = 0.002) (Table 1). When the clinical features associated with MuSK MG were analyzed by multivariate logistic regression model, female sex (OR 10.01; 95% CI 2.42–42.31), ocular form at onset (OR 5.17; 95% CI 1.08–24.79), experience of myasthenic crisis (OR 4.51; 95% CI 1.18–17.32) and high MGCS score (OR 1.09; 95% CI 1.00–1.18) were significantly associated with the diagnosis with MuSK MG (Table 2).

**Table 1:** Comparisons between MuSK MG, double seronegative generalized MG (dSNMG) and motor neuron disease (MND) groups

	MuSK MG (*n* = 27)	dSNMG (*n* = 62)	MND (*n* = 30)	*P* values	
				MuSK MG vs. dSNMG	MuSK MG vs. MND
Onset age, mean (years) [range]	48.2 [21–71]	48.0 [20–82]	62.4 [34–78]	> 0.1	< 0.001
Female, % (*n*)	85.2 (23/27)	43.5 (27/62)	46.7 (14/30)	< 0.001	0.005
Ocular form at onset, % (*n*)	25.9 (7/27)	12.9 (8/62)	0.0 (0/30)	> 0.1	0.004
MuSK Ab positive					
ELISA, % (*n*)	81.5 (22/27)	0.0 (0/62)	6.7 (2/30)	< 0.001	< 0.001
CBA, % (*n*)	92.6 (25/27)	0.0 (0/62)	NA	< 0.001	NA
RIPA, % (*n*)	82.4 (14/17)	0.0 (0/34)	NA	< 0.001	NA
RNST (abnormal decrements), % (*n*)	77.8 (21/27)	66.1 (39/59)	41.4 (12/29)	> 0.1	0.007
Pharmacological response, % (*n*)	66.7 (4/6)	75.0 (15/20)	23.5 (4/17)	> 0.1	> 0.1
Thymic hyperplasia or thymoma, % (*n*)	12.0 (3/25)	15.7 (8/51)	0.0 (0/23)	> 0.1	> 0.1
MGFA classification at presentation					
≥ 3, % (*n*)	22.2 (6/27)	19.4 (12/62)	28.6 (8/28)	> 0.1	> 0.1
B classification, % (*n*)	70.4 (19/27)	40.3 (25/62)	78.6 (22/28)	0.012	> 0.1
Current MGFA classification					
≥ 3, % (*n*)	14.8 (4/27)	16.1 (10/62)	NA	> 0.1	NA
B classification, % (*n*)	66.7 (18/27)	30.6 (19/62)	NA	0.002	NA
Myasthenic crisis or mimics, % (*n*)	40.7 (11/27)	11.3 (7/62)	3.8 (1/26)	0.003	0.002
MGCS, mean [range]	9.8 [0–33]	6.1 [0–24]	10.1 [0–26]	0.046	> 0.1

*Ab* Ab, *CBA* cell-based assay, *dSNMG* double seronegative generalized myasthenia gravis, *ELISA* enzyme-linked immunosorbent assay, *MGCS* myasthenia gravis composite scale, *MGFA* myasthenia gravis foundation of America, *MND* motor neuron disease, *MuSK* muscle specific tyrosine kinase, *NA* not available, *RIPA* radioimmunoprecipitation assay, *RNST* repetitive nerve stimulation test

**Table 2:** Logistic regression analysis of clinical features associated with MuSK MG in comparison to double seronegative generalized MG (dSNMG) and motor neuron disease (MND)

Variables	Univariate analysis			Multivariate analysis		
	OR	95% CI	*p* value	OR	95% CI	*p* value
MuSK MG vs. dSNMG						
Age at onset	1.00	0.97–1.03	0.952	–	–	–
Sex	7.45	2.30–24.12	0.001	10.01	2.42–41.31	0.001
Ocular form at onset	2.36	0.76–7.36	0.138	5.17	1.08–24.79	0.040
Abnormal RNST result	1.80	0.63–5.16	0.277	–	–	–
Pharmacological response: positive	0.67	0.09–4.81	0.688	–	–	–
Thymic hyperplasia or thymoma	0.73	0.18–3.04	0.669	–	–	–
MGFA at presentation ≥ 3	1.19	0.39–3.59	0.757	–	–	–
MGFA B classification at presentation	3.52	1.33–9.27	0.011	3.30	0.94–11.56	0.061
Myasthenic crisis	5.40	1.80–16.21	0.003	4.51	1.18–17.32	0.028
MGCS	1.08	1.01–1.16	0.022	1.09	1.00–1.18	0.046
MuSK MG vs. MND						
Age at onset	0.92	0.87–0.97	0.001	0.92	0.86–0.98	0.016
Sex	6.57	1.83–23.67	0.004	15.47	1.35–176.59	0.028
Abnormal RNST result	4.96	1.54–15.98	0.007	8.67	1.27–58.99	0.027
Pharmacologic response: positive	4.80	0.66–35.20	0.123	–	–	–
MGFA at presentation ≥ 3	0.71	0.21–2.43	0.590	–	–	–
MGFA B classification at presentation	0.65	0.19–2.20	0.487	–	–	–
Myasthenic crisis	17.19	2.02–146.25	0.009	115.12	3.50–3783.92	0.008
MGCS	1.00	0.93–1.07	0.881	–	–	–

The variables of ocular form at onset and thymic hyperplasia or thymoma were excluded from the latter analysis because none of MND patients had neither ocular form at onset nor thymic hyperplasia/thymoma

*MGCS* myasthenia gravis composite scale, *MGFA* myasthenia gravis foundation of America, *RNST* repetitive nerve stimulation test

Clinical features of the seven patients with discordant results on different assays for MuSK Ab were summarized in Table 3. The 5 ELISA-negative but CBA-positive patients mostly showed predominantly bulbar symptoms and good treatment response to immunosuppressive agents, suggesting that their ELISA results were likely to be false-negative. Meanwhile, two patients were marginally positive for MuSK Ab in ELISA but negative in CBA (RIPA negative in one, and not done in the other), raising the possibility of false-positive (ELISA) or false-negative (CBA/RIPA) which may arise from various sources (including the sensitivity/specificity issue of the assay itself and others such as sample quality). One of these was a young female in her twenties who presented with diplopia, dysarthria and swallowing difficulty. There were abnormal decrements of compound muscle action potentials (CMAP) in deltoid muscle on RNS, and positive result in neostigmine test. Her symptoms improved with intravenous immunoglobulin and prednisolone treatment. The other middle-aged woman presented with bulbar predominant symptoms which progressed to myasthenic crisis and improved with immunosuppressive treatment.

**Table 3:** Clinical features of the patients with discordant results on different assays (ELISA, CBA, RIPA) for anti-MuSK antibody

Pt. no	Sex/age	Anti-MuSK Ab			Disease duration (months)	MGFA classification (onset)	MGFA classification (current)	RNST abnormality	Thymus abnormality (CT)	Pharmacologic test	Myasthenic crisis	MGFA PIS
		ELISA (unit/ml)	CBA (score)	RIPA (cpm)								
1	M/71	Neg	Pos (1.00)	Neg	13	IIIb	IIb	Yes	No	Pos	Yes	Improved
3	F/28	Pos (0.81)	Neg	Neg	7	IIb	IIb	Yes	No	Pos	No	Improved
74	F/43	Pos (1.47)	Neg	–	55	IIb	IIb	No	No	Pos	Yes	Improved
80	F/28	Neg	Pos (2.75)	Pos (1154)	12	IIb	IIb	Yes	No	–	Yes	Improved
86	F/56	Neg	Pos (1.00)	Neg	12	IIb	IIb	No	No	–	No	MM
183	M/67	Neg	Pos (3.50)	–	21	IIb	IIIb	Yes	No	Pos	No	Worsened
188	F/51	Neg	Pos (4.00)	–	15	IIb	V	Yes	No	–	Yes	Worsened

*Ab* antibody, *CBA* cell-based assay, *cpm* counts per min, *ELISA* enzyme-linked immunosorbent assay, *F* female, *LEMS* Lambert-Eaton myasthenic syndrome, *M* male, *MGFA* myasthenia gravis foundation of America, *MM* minimal manifestation, *MuSK* muscle specific tyrosine kinase, *PIS* post-intervention status, *Pos* positive, *Pt.no* patient number, *RIPA* radioimmunoprecipitation assay, *RNST* repetitive nerve stimulation test

## Discussion

In this study, we evaluated the diagnostic accuracy of MuSK-ELISA in a large multi-center cohort of 89 AChR Ab-negative generalized MG, and confirmed that the ELISA results are consistent with those of CBA and RIPA in the vast majority of patients with Cohen’s kappa of 0.80 and 0.90, respectively. We also found significant correlations of anti-MuSK Ab concentrations in ELISA with CBA scores and RIPA values. Taken together with the high specificity of MuSK-ELISA in our disease controls, our results support a diagnostic utility of MuSK-ELISA in clinical practice.

This study highlights real-world challenges and clinical pitfalls in a diagnosis of seronegative generalized MG, particularly without the aid of serological testing. Though initially suspected of AChR Ab-negative generalized MG, a final diagnosis in a considerable portion of the patients (56/160, 35.0%) turned out to be not MG. The most common differential diagnosis in our cohort was motor neuron disease, particularly of bulbar onset. It is noteworthy that there were remarkably overlapping clinical and electrophysiological features between MuSK MG and motor neuron disease at presentation. More than two thirds of MuSK MG patients (70%) showed predominantly bulbar involvement, often with tongue and facial muscles atrophy. Meanwhile, in about 40% of motor neuron disease patients, RNS test revealed abnormal (> 10%) decrements in CMAP amplitudes, reflecting the neuromuscular junction transmission defects. In bulbar-onset motor neuron disease, symptoms may be confined to the bulbar region for several months or even years before wider generalization often with a lack of EMG findings of subclinical lower motor neuron involvement at limb muscles. These clinical and electrodiagnostic overlaps and pitfalls might account for partly at least why motor neuron disease constituted such a large proportion of differential diagnoses in our cohort. Of note, however, the motor neuron disease patients were found to be significantly different from those with MuSK MG in that they were significantly older at symptom onset, the sex ratio was not biased to female, none had ocular manifestations, and the myasthenic crisis or mimics were very rare.

Clinical features of our MuSK MG patients agreed well with the known characteristics, including female preponderance, predominantly bulbar involvement, rare thymic pathology, rapid progression and frequent myasthenic crisis. Notably, some of these features (female preponderance, predominant bulbar impairment, frequent crisis, and greater severity) were also significantly over-represented in MuSK MG compared to double seronegative MG in our cohort. Further research to discover new self-antigens and autoantibodies would be required to understand the composition and clinical characteristics of MG subtypes within this group.

The overall proportion of ocular form at onset was relatively low in our cohort of AChR Ab-negative generalized MG patients. Unexpectedly, however, the ocular form at onset was significantly associated with the diagnosis of MuSK MG when compared to double seronegative generalized MG. Although the involvement of extra-ocular muscles was initially thought to be rare in MuSK MG, a recent study of Italian patients showed that it was frequent as in AChR MG, reporting it as the first manifestation in almost 60% of patients [17]. Given the large variation across studies, it is likely that the ocular manifestations in MuSK MG may be subtle or atypical and therefore require careful attention to notice [17–19].

This study was retrospective, and the results might have been affected by possible selection bias and variation in clinical practice among participating physicians. Both ELISA and CBA were performed in all 89 patients, but RIPA in a subset of patients (*n* = 51). This limitation of the study was due to the practical difficulties of obtaining clinical serum samples from a large number of varying sources. In line with previous works [8, 10, 16], however, we confirmed a very high agreement between the results of CBA and RIPA in these 51 patients, which may support the validity of our findings. Diagnostic certainty of double seronegative MG may be another limitation of this study. It may be related in part to the sensitivity shortfall of ancillary tests and a lack of the formal established diagnostic criteria for seronegative MG. While we screened an initial cohort of patients with a high index of suspicion for seronegative generalized MG, we noted that the level of suspicion varied among cases and making a clinical diagnosis are often challenging particularly at initial presentation. Indeed, there were no abnormal findings consistent with MG in either RNS or pharmacologic test in 27% (17/62) of double seronegative MG patients. Single-fiber EMG was rarely performed in our group of patients, probably because it is technically demanding and non-specific. Instead of excluding these patients, we decided to make a diagnostic judgment based on disease course and treatment response during clinical follow-up of at least 6 months. To elucidate the sensitivity and specificity of diagnostic tests in seronegative MG, we propose that formal consensus in clinical diagnostic criteria should be established. Lastly, the specificity of MuSK ELISA was assessed only in disease controls in this study. Although the specificity of CBA and RIPA was examined in a subset of disease controls (12/63), the result was well in line with previous works supporting the well-established validity of these assays [8, 10, 16].

Although we did not present the sensitivity of MuSK-ELISA, it can be inferred to be rather lower than that of CBA from direct comparisons of the results. As for the positive predictive value of MuSK-ELISA, it is proportional not only to the sensitivity but also to the prior probability for the diagnosis. We’ve encountered many instances in which patients with bulbar onset MND were referred for MuSK ELISA mainly based on the complaints of fatigable weakness and a decremental response in RNS test. Given the caveat of false-positives in MuSK ELISA, our results indicate that the prior probability of MuSK MG should be adjusted with careful consideration of the diagnostic pitfalls. Meanwhile, it also should be emphasized that the negative result of MuSK-ELISA should not preclude the diagnosis of MuSK MG. Tests with higher sensitivity should be considered when MuSK MG is clinically suspected with exclusion of alternative diagnoses.

In conclusion, we confirmed the diagnostic accuracy of MuSK-ELISA in a large cohort of AChR Ab-negative generalized MG patients. Our results highlight the clinical pitfalls in making a diagnosis of MuSK MG, and support a diagnostic utility of MuSK-ELISA in clinical practice particularly where either RIPA or CBA is not available.
